# The effects of ventilator settings, nebulizer and exhalation port positions on albuterol delivery during noninvasive ventilation: an in vitro study

**DOI:** 10.1186/2197-425X-3-S1-A169

**Published:** 2015-10-01

**Authors:** Y Sutherasan, P Raimondo, L Ball, V Caratto, E Sanguineti, M Ferretti, P Pelosi

**Affiliations:** IRCCS AOU San Martino-IST, Department of Surgical Sciences and Integrated Diagnostics, University of Genoa, Genoa, Italy; Faculty of Medicine, Ramathibodi Hospital, Mahidol University, Division of Pulmonary and Critical Care Medicine, Department of Medicine, Bangkok, Thailand; Università degli Studi di Foggia, Dipartimento di Anestesia, Rianimazione e Terapia Intensiva, Foggia, Italy; University of Genoa, Department of Chemistry and Industrial Chemistry, Genoa, Italy; SPIN-CNR, Genoa, Italy

## Introduction

Noninvasive ventilation (NIV) has shown benefit in term of decrease in mortality in COPD exacerbation. The nebulized bronchodilators are mostly prescribed as the role of reversible of obstruction in these patients. However, there have been few studies demonstrating the factors those affect the aerosol delivery during NIV.

## Objectives

We aimed to investigate the effect of different positions of exhalation port and nebulizer, ventilator setting delivered by NIV on the amount of aerosol bronchodilator delivery during simulated spontaneous breathing.

## Methods

**Figure 1:**
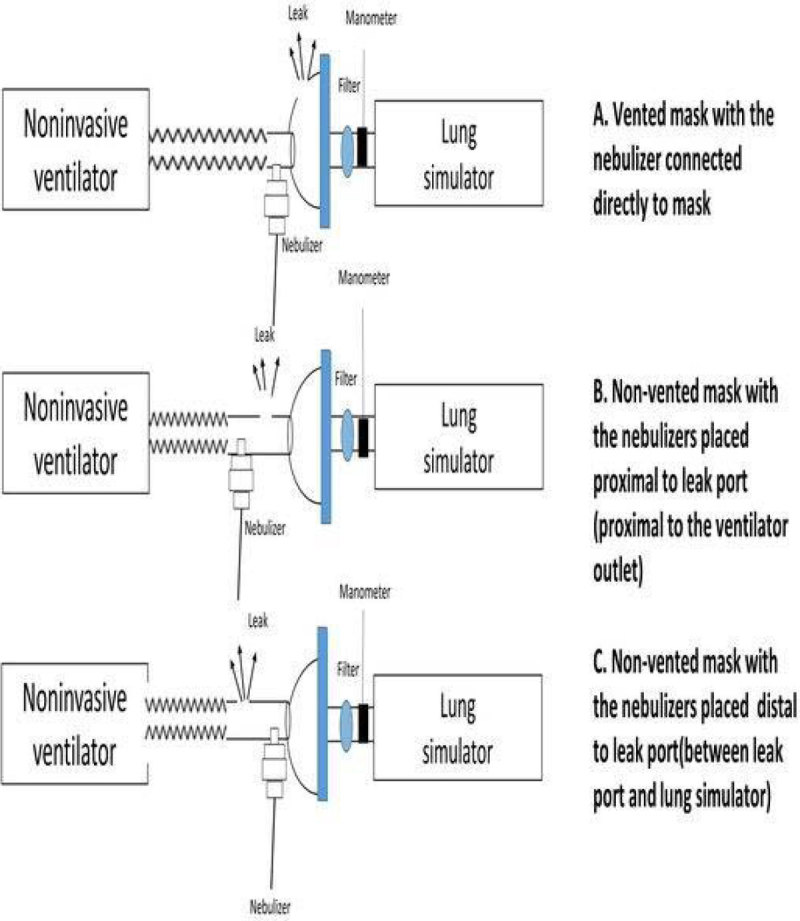
**Schematic diagram of experimental procedure and different types of mask and bebulizer positions.**

A noninvasive ventilator (Covidien Puritan Bennett® 560^TM^) was connected to a lung model that simulated spontaneous breathing. The noninvasive ventilator was set for the spontaneous mode, trigger 2 L/min, back up respiratory rate 13/min and target tidal volume of 0.4 L. A nebulizer was filled with 5 mg of albuterol in 3 ml of solution driven with 8 L/min oxygen source. The experimental settings varied in the following features: 1)Bi-level positive-airway-pressure ventilator (BIPAP) settings with the inspiratory positive airway pressure and expiratory positive airway pressure (EPAP) of 10/5, 15/10, 15/5, and 20/10 and continuous positive airway pressure of 5 and 10 cmH_2_O and 2)Two mask types were used: one, in which the leak port was incorporated into the mask(vented mask) and the nebulizer connected directly to mask(Figure 1A) and another in which the leak port was incorporated into the circuit(non-vented mask)(Figure 1B and 1C).In non-vented mask group, the nebulizers were placed either proximal to leak port (Figure 1B) or distal to leak port (between leak port and lung simulator)(Figure 1C).

Albuterol was collected with a filter and measured the percent amount delivered by infrared spectrophotometry.

## Results

**Figure 2:**
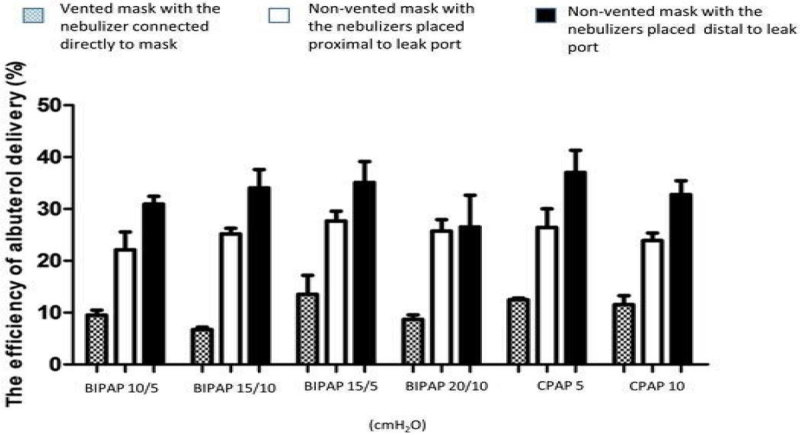
**Efficiency of albuterol delivery during NIV system at different IPAP and EPAP levels and nebulizers position.**

Albuterol delivery in NIV varied between 6.73 ± 0.44% to 37.01 ± 4.31% of the nominal dose. There were the significant difference in albuterol delivery between the mask types and nebulizer positions under 4 combinations of BIPAP levels and 2 levels of CPAP (p < 0.001)(Figure 2)

The highest albuterol delivery was observed with the nebulizer operating at the distal position. The system in which the nebulizer is connected directly to the vented mask had the lowest aerosol delivery (p < 0.001) (Figure 2). Under different levels of BIPAP setting, the higher levels of EPAP show significantly decrease in albuterol delivery (21.15 ± 10.58%with EPAP 10 cmH_2_O vs. 23.15 ± 9.73%with EPAP 5 cmH_2_O, p < 0.001).

## Conclusions

Albuterol delivery with NIV was affected by the position of leak port and nebulizer and the ventilator setting. We recommend placing the nebulizer operating at the distal position to the leak port.
